# Cohort Profile: The Socioeconomic Consequences in Adult Life After Childhood Cancer in Scandinavia (SALiCCS) Research Programme

**DOI:** 10.3389/fonc.2021.752948

**Published:** 2021-11-26

**Authors:** Friederike Erdmann, Line Elmerdahl Frederiksen, Hanna Mogensen, Camilla Pedersen, Luzius Mader, Mats Talbäck, Andrea Bautz, Elli Hirvonen, Anniina Kyrönlahti, Liisa Maria Korhonen, Henrik Hasle, Nea Malila, Laura-Maria Madanat-Harjuoja, Maria Feychting, Jeanette Falck Winther

**Affiliations:** ^1^ Childhood Cancer Research Group, Danish Cancer Society Research Center, Copenhagen, Denmark; ^2^ Division of Childhood Cancer Epidemiology, Institute of Medical Biostatistics, Epidemiology and Informatics (IMBEI), Johannes Gutenberg University Mainz, Mainz, Germany; ^3^ Unit of Epidemiology, Institute of Environmental Medicine, Karolinska Institutet, Stockholm, Sweden; ^4^ Institute of Social and Preventive Medicine (ISPM), University of Bern, Bern, Switzerland; ^5^ Institute for Statistical and Epidemiological Cancer Research, Finnish Cancer Registry, Helsinki, Finland; ^6^ New Children’s Hospital, University of Helsinki and Helsinki University Hospital, Helsinki, Finland; ^7^ Department of Paediatrics, Aarhus University Hospital, Aarhus, Denmark; ^8^ Dana Farber Cancer Institute/Boston Children’s Cancer and Blood Disorders Center, Harvard Medical School, Boston, MA, United States; ^9^ Department of Clinical Medicine, Faculty of Health, Aarhus University and Aarhus University Hospital, Aarhus, Denmark

**Keywords:** childhood cancer survivors, survivorship, social and socioeconomic outcomes, family life, register-based research, Denmark, Finland, Sweden

## Abstract

**Introduction:**

The growing number of survivors of childhood cancer, with many years of life ahead, demonstrates the increasing clinical and public health relevance of investigating the risks of social and socioeconomic impairment after a childhood cancer diagnosis and the life-saving treatment. To enrich understanding of the mental, social and socioeconomic difficulties that childhood cancer survivors may face during their life-course, identify particularly vulnerable survivors and overcome the limitations of previous research, we initiated the Socioeconomic Consequences in Adult Life after Childhood Cancer in Scandinavia (SALiCCS) research programme.

**Methods:**

This Nordic cross-border research programme is a collaboration between the Danish Cancer Society, the Finnish Cancer Registry and Karolinska Institutet to investigate a broad range of mental, social and socioeconomic conditions in long-term childhood cancer survivors in Denmark, Finland and Sweden. SALiCCS is based on a registry-based matched cohort design, comprising five-year survivors of cancer diagnosed at ages 0–19 years (1971–2008 in Denmark, 1971–2009 in Finland, 1971–2011 in Sweden), age-, sex- and country-matched population comparisons and sibling comparisons who were followed over time. Outcomes of interest included mental disorders, educational achievements, employment and profession, family life and the need of social security benefits. Individual-level data linkage among various national registries provided the data for the research programme.

**Results:**

The SALiCCS core population comprises 21,292 five-year survivors, 103,303 population comparisons and 29,644 siblings as a second comparison group. The most common diagnoses in survivors were central nervous system tumours, leukaemias and lymphomas.

**Discussion:**

SALiCCS is the largest, most comprehensive population-based research initiative in this field, based on high-quality registry data with minimal risk of bias. The findings will be informative for evidence-based survivorship care targeting not only somatic late effects but also psychosocial impairments.

## Introduction

Childhood cancer is of increasing public health concern, as approximately 35,000 new cases are diagnosed yearly in children and adolescents in Europe, and about 500,000 European Union citizens are childhood cancer survivors, with complex needs for medical and psycho-social care (1). Although a growing body of research has addressed a broad range of potential risk factors, the aetiology of most childhood cancers is still largely unknown (2, 3). With remarkable advances in diagnostics and treatment (4, 5), the five-year survival after childhood cancer has improved from 30% in the 1960s to more than 80% nowadays in most of Europe (6–8). As a result of the increasing survival and lack of primary preventive measures (2, 3, 9), the number of childhood cancer survivors in society is growing steadily. This growing population is at risk of long-term health consequences (i.e. late effects) induced by the cancer or the intensive treatment at a young age (8, 10–12). Although many survivors are well after therapy, a wide spectrum of long-term adverse health consequences in childhood cancer survivors has been described (8, 12–18), indicating higher risks of a broad range of somatic and mental late effects, including second cancers (8, 12, 17, 18), higher overall mortality rates (12, 16), severe chronic health conditions (8, 12–15), mental disorders (19, 20) and use of antidepressants (21).

The experience of cancer during childhood and adverse somatic or mental health conditions may also have consequences for social and family life and for socioeconomic achievement later in life. Previous research has shown that childhood cancer survivors are at increased risk of several adverse socioeconomic and social outcomes, including scholastic difficulties, such as requiring special education or attending learning disability programmes, lower levels of attained education and lower income than their peers (22). There are only few studies on the uptake of social security benefits in survivors, and almost all are from the Nordic countries. Increased uptake of various social security benefits by survivors was reported consistently (22). Empirical observations on employment and occupation are less conclusive (22, 23), with heterogeneous findings from Europe (22, 23) and a consistently higher risk of unemployment among survivors in studies from Canada and the USA (23–25).

The current evidence is limited by methodological shortcomings. Most previous research is based on self-reported information from surveys and are thereby susceptible to non-participation, which might have affected the outcomes. Further, many studies included survivors of only one or a few specific childhood cancer types, did not involve repeated measurements of social and socioeconomic outcomes throughout the life-course or suffered from substantial loss to follow-up. Further limitations of previous studies include insufficient sample size of survivors and short follow-up. The mechanisms that lead to adverse social and socioeconomic conditions, especially in vulnerable subgroups of survivors, are still poorly understood (22, 26), and better knowledge would be of significant importance for developing interventions and supportive strategies for these vulnerable groups.

The Socioeconomic Consequences in Adult Life after Childhood Cancer in Scandinavia (SALiCCS) research programme was initiated to address these gaps and enrich understanding of the impairments and social and socioeconomic difficulties that survivors of childhood cancer may face during their life-course. This Nordic research programme is a collaboration among the Danish Cancer Society Research Center, the Finnish Cancer Registry and Karolinska Institutet in Sweden.

### The Welfare Systems of Denmark, Finland, and Sweden

In 2020, Denmark, Finland and Sweden had populations of 5.8, 5.5 and 10.3 million, respectively (27). The Nordic countries are well known for their generous welfare systems (28), with the core principles of solidarity and universalism and the overall aim of providing equal access for all citizens to welfare services, including health care, education and social security benefits. Decommodification in the three countries ensures that such services are provided independently of an individual’s affiliation to the labour force (28). The welfare services are tax-funded and, as a result, Denmark, Finland and Sweden have some of the highest tax revenues in the world of above 40% of the gross domestic product (GDP) (29). In general, the welfare services provided in these countries result in a high standard of living, demonstrated for instance by high life expectancy (30).

The welfare systems of the three countries are largely comparable. Citizens are entitled to an education free of charge, from primary schooling, which is compulsory, to advanced tertiary educational levels (31). Additionally, students enrolled in tertiary education, such as college or university, may receive direct financial support and loans from the government. The unemployment rates in Denmark, Finland and Sweden are low (32) (from 5.7% to 8.8% in 2017), and few citizens rely on income support from the government (i.e. social security benefits) (32). Increasing numbers of women in the Nordic countries have entered the workforce over the past six decades (33), and the active labour force has almost equal gender distribution (32). This is to some extent enabled by supportive arrangements such as state-subsidised childcare provision, generous parental leave schemes and, often, flexible working hours (34, 35).

In general, all citizens of the Nordic countries have equal access to government-subsidized primary health care services provided by general practitioners and specialised health care in hospitals or provided by specialist physicians. Out-of-pocket expenses and reimbursement schemes vary, however, in the three countries (36–38). The health care services in all three countries also cover all costs directly related to the diagnosis and treatment of childhood cancer and for the vast majority of health care for any late effects. In Denmark and Sweden, more than 80% of all health care expenditure is covered by the public tax-financed system (36, 37, 39). In Finland, total public coverage of health care expenditures is also about 80%, funded primarily through taxation (around 60%) but also through health insurance contributions paid to the tax administration (38).

### Childhood Cancer Treatment in the Nordic Countries

As childhood cancers are a heterogeneous group consisting of very different diseases, survival and developments in survival over time differ widely by cancer type (6). Since the 1970s, as a result of advances in molecular tumour biology, imaging, pharmacology, risk grouping and treatment combinations, overall five-year survival rose from 30% in the pre-chemotherapeutic era to more than 80% nowadays (6). Early collaborative clinical research to identify effective therapy for children with cancer dates back to the 1950s, when children with acute lymphoblastic leukaemia were some of the earliest participants in clinical trials of new drugs for cancer treatment (5). Participation in clinical trials is today considered the standard of paediatric cancer care, and a large number of children in Europe and North America are enrolled in protocols (4, 40–42) developed by collaborative study groups, such as the Nordic Society of Paediatric Haematology and Oncology (NOPHO) (4, 40, 41).

Current therapy for some malignancies is highly intensive, and, while survival has gradually increased, the risk of treatment-related acute toxicity and late effects may also be increasing. The aim of many current protocols is to identify subgroups of patients for whom the intensity of therapy can be reduced to decrease toxicity.

Collaborative clinical trials to standardise childhood leukaemia treatment protocols in all Nordic countries began in 1981 (4, 40, 43), and since 1992 almost all children with leukaemia have been treated with the standardised NOPHO protocols. Treatment for lymphomas and solid tumours has been similarly standardised and is based mainly on the protocols of international collaborative study groups with NOPHO participation.

### Aims and Objectives of the SALiCCS Research Programme

The SALiCCS research programme is a Nordic register-based cohort study of social and socioeconomic consequences in long-term survivors of childhood cancer in Denmark, Finland and Sweden. SALiCCS is based on the Nordic research programme Adult Life after Childhood Cancer in Scandinavia (ALiCCS), which investigates late effects of cancer therapy in children to better understand the risk and mechanisms of treatment-induced somatic disease (44). Data from ALiCCS are enriched by longitudinal information on mental disorders and social and socioeconomic outcomes after a childhood cancer diagnosis. With this strategy, the overarching goal of SALiCCS is to identify groups of childhood cancer survivors for whom early intervention would minimise later mental disorders and adverse social and socioeconomic consequences of the cancer and its treatment.

The main objectives of the SALiCCS research programme are:

- to ascertain hospital contacts for mental disorders in childhood cancer survivors;
- to examine how survivors of childhood cancer transition from childhood to adulthood by determining the following social and socioeconomic conditions and attainments: scholastic achievements, attained educational level and educational delays, income, employment, occupational position and professional attainment, leaving the parental household to live independently, cohabitation with a partner, getting married and founding a family; and
- to assess the socioeconomic burden of childhood cancer and treatment on survivors by determining the uptake of social security benefits, such as unemployment benefits, social assistance, sickness allowance, disability pension and rehabilitation benefits.

## Material and Methods

### Design and Research Setting

**Figure 1:**
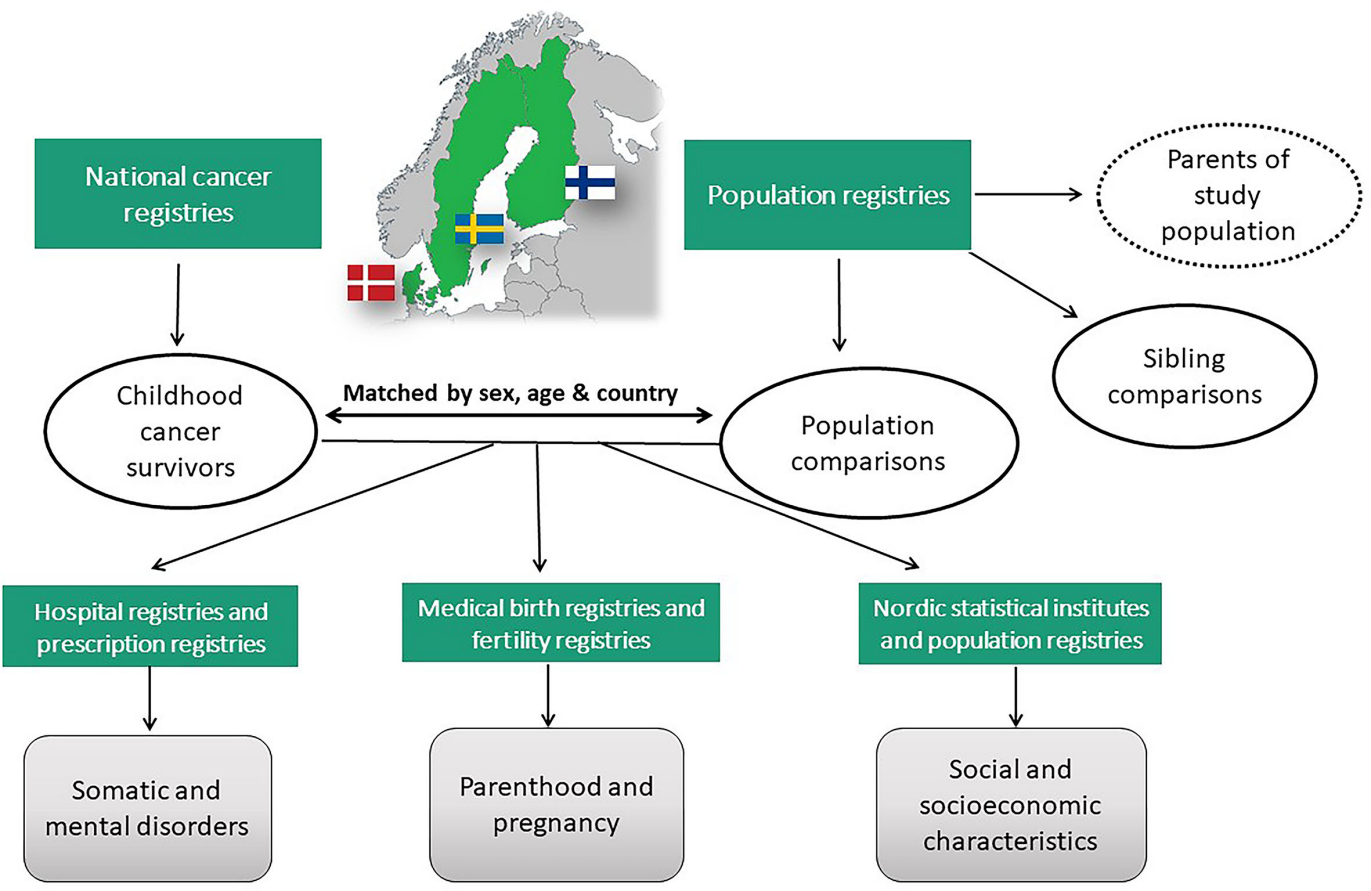
Overview of the SALiCCS study design and data sources.

The SALiCCS research programme is based on a registry-based matched cohort design. Denmark, Finland and Sweden have civil registration systems with numerous national administrative registries (45–47) that contain individual-level data in various fields, including cancer diagnoses (48), hospitalisation for somatic and mental disorders (49–52), vital status (46, 53), emigration and immigration (45, 46), perinatal and birth characteristics (54–56) and socio-demographic and socioeconomic characteristics (57–64). Nordic citizens are assigned a unique personal identification number (Denmark since 1968, Finland since 1964 and Sweden since 1947) that is used in all national registries, enabling accurate linkage of information among registries (45–47, 65). National legislations permit and supports registry-based research. Data linkage among the registries is the basis of the SALiCCS research programme. Data from all three countries are collected and harmonized to enable pooled analyses (see section 2.5 for information on data access and data protection). **Figure 1** gives an overview of the SALiCCS study design and the registers used.

The infrastructure of the population-based registers in the Nordic countries, with longstanding, high-quality, comprehensive health, socio-demographic and socioeconomic data, are an ideal, unique basis for large-scale epidemiological studies of childhood cancer survivorship. None of the Nordic countries, however, has a sufficiently large population to provide adequate statistical power for a detailed assessment of social and socioeconomic outcomes in childhood cancer survivors in a life-course perspective, particularly not for determining the underlying mechanisms of adverse social and socioeconomic outcomes and identifying particularly vulnerable groups of survivors. Combination of data from several Nordic countries is required for such purposes. As the Nordic countries have longstanding, largely standardised diagnostic and treatment procedures and similar welfare systems, it was considered reasonable to combine data on childhood cancer survivors across Nordic countries.

### Study Population

The SALiCCS core population comprises all five-year survivors of a first childhood cancer (including non-malignant central nervous system tumours) diagnosed at ages 0–19 years in Denmark (1971–2008), Finland (1971–2009) and Sweden (1971–2011) (**Table 1** and **Figure S1**). For individual SALiCCS studies however, also more broadly defined criteria for the study population may be applied (e.g. 1-year survival as eligibility criteria or including all childhood cancer cases). We identified childhood cancer cases from the respective nationwide cancer registries, which are of excellent quality and high completeness (48).

**Table 1:** Study period and data availability of parental information and information about the index subjects in the SALiCCS research programme by country.

	Denmark	Finland	Sweden
**Study period of cancer diagnosis/reference**	1971-2008	1971-2009	1971-2011
**End of follow-up**	11 Aug 2017	31 Dec 2014	31 Dec 2016
**Time period with available sociodemographic and socioeconomic information**			
Region of residence	1980-2017	1936-2016	1968-2016
Educational achievements	1970-2016	1970, 1975, 1980, 1985, 1987-2016	1960, 1963-2016^a^
Employment	1980-2017	1970, 1975, 1980, 1985, 1987-2016	1960, 1965, 1970, 1975, 1980, 1985, 1990-2015
Occupational position	1993-2017	1990, 1993, 1995, 2000, 2004-2015	1960, 1970, 1975, 1980, 1985, 2001-2015
Disposable income	1980-2018	1995-2016	1970^b^, 1975, 1985, 1990-2015
Social security benefits	1980-2016 (differs between benefit types)	1985-2016 (differs between benefit types)	1990-2015 (differs between benefit types)
Marital status	1968-2017	1970-2016	1960, 1965, 1968-2016
Cohabitation status	1980-2017	1987-2016	1970, 1975, 1980, 1985, 1990-2015^d^
Parenthood	1968-2017	1971-2016	1961-2016
**Time period with available information on hospital contacts for somatic and psychiatric care and prescribed drugs**			
Somatic diseases	1977-2017	1969-2014	1964^c^-2016
Mental disorders	1969-2017	1975-2014	1973^c^ -2016
Prescribed drugs	1995-2017	1995-2016	2005-2017

a Differences in the availability of educational information for the early time period.

b Net income is used in 1970 as disposable income was not available.

c Nationwide since 1987.

d 1990-2015: Restricted to cohabitating couples with common children.

The cancer diagnoses were classified according to the International Classification of Childhood Cancer, in which tumours are classified into 12 major diagnostic groups and detailed subgroups according to the nomenclature of the International Classification of Diseases – Oncology (66–69).

We established two independent comparison groups. We randomly sampled five population-based comparisons per survivor from the populations of Denmark, Finland and Sweden from the national population registries. Survivors and population comparisons were individually matched by year of birth, sex and country of residence (and municipality of residence in Sweden) (**Figure 1** and **Figure S1**). The second comparison group constitutes all biological full and half siblings as well as adopted (except in Finland) siblings of the childhood cancer survivors, defined as having either the same (biological or adoptive) mother or father. One sibling could serve as a sibling comparison for several survivors if more than one child in the same family was a childhood cancer survivor. We included only siblings with a maximum age difference of 10 years in order to allow meaningful comparisons of outcomes between siblings and corresponding childhood cancer survivors, leaving the possibility to reduce the age difference even more for some outcomes when relevant. Sibling and population comparisons had to be alive and not lost to follow-up five years after the reference date, and cancer-free until the age of 20 years to be eligible as comparisons. The reference date was defined as the date of diagnosis of cancer in the corresponding matched survivor for population comparisons and, for siblings, as the date on which the sibling was of the same age as the corresponding survivor at cancer diagnosis.

As a cancer predisposition syndrome may confound associations with mental, social and socioeconomic outcomes, we excluded individuals with Down syndrome, neurofibromatosis or tuberous sclerosis, resulting in a final SALiCCS core population of 21,292 five-year childhood cancer survivors, 103,303 population comparisons and 29,644 siblings (**Figure S1**).

### Follow-Up, Social and Socioeconomic Outcomes and Information on Potential Mediators and Confounders

The childhood cancer survivors and their comparisons were followed from five years after the cancer diagnosis or reference date until death, first emigration, loss to follow-up or end of follow-up, whichever came first. Information on end of follow-up varied somewhat by outcome data, depending on the registry used as the data source (**Table 1**).

For childhood cancer survivors and their comparisons, we obtained comprehensive information on highest attained education, scholastic achievements, educational delays and other educational information, individual and household disposable income, labour market affiliation, occupation position and the uptake of various social security benefits, including annual unemployment, sickness and disability benefits, social assistance and rehabilitation allowances from the social registries administered by the Nordic statistical institutes. “Disposable income” refers to annual individual and household income after taxes, including social security benefits.

Information on family structure, including cohabitation and marriage, place of residence, parenthood and other socio-demographic information, was obtained from the population registries, while data on birth characteristics were obtained from the medical birth registers, which contain mandatory, regularly updated reports on all births in the respective countries. From the national patient registries, we received comprehensive histories of hospital admissions (including outpatient contacts) for somatic and mental disorders and the respective discharge diagnoses. We obtained information on prescribed drugs from the nationwide prescribed drug registries, whereby prescription data from Finland was limited to contraceptive medications, antidepressants, pain killers, and psychiatric drugs.

Apart from diagnostic data from the national cancer registries, clinical and treatment information was overall sparse. The Finnish Cancer Registry included limited and non-validated treatment information on surgery, radiotherapy and chemotherapy (with incomplete coverage). The treatment information is given on binary level for curative, palliative or unknown intention (48). For Denmark and Sweden, some clinical and treatment data was available from the Swedish Childhood Cancer Registry and from the NOPHO database, however only for leukaemia survivors and for a limited follow-up period.

### Parental Information

We collected basic socio-demographic and socioeconomic information for the biological and adoptive parents (for Finland only for biological parents) of the childhood cancer survivors, the population comparisons, and the sibling comparisons. Furthermore, to account for the increasing number of reconstituted families in the Nordic countries, we also defined the “social parents” for the Danish and Swedish SALiCCS populations, for whom we collected the same socioeconomic and socio-demographic information as for biological parents. In Denmark, social parents were defined as individuals living at the same address with the index person the year before the reference year, at least 16 years older and not a full or half sibling of the index person. In Sweden, social parents were defined as individuals registered by Statistics Sweden as a parent or guardian in the same household in which the index person was defined, the year before the reference date. Social parents could be identified from 1980 onwards in Denmark and from 1990 onwards in Sweden. Corresponding information was not collected for the Finnish SALiCCS population. 

### Data Access, Data Protection and Other Ethical Considerations

The SALiCCS research programme has been approved by Statistics Denmark, the Regional Ethical Review Board in Stockholm, Sweden (dnr 2016/25-31/5, 2016/1561-32, 2017/1656-32, 2017/1990-32, 2017/2340-32, 2018/1165-32), Findata (Dnro THL/5543/14.06.00/2020) prolonging the former approvals by the National Institute for Health and Welfare and Social Insurance (KELA) and Statistics Finland (TK-53-394-17) in Finland. For the European Union General Data Protection Regulation (GDPR), the SALiCCS project is listed in a local archive (2018-DCRC-0044) at the Danish Cancer Society Research Center, which provides an accurate, updated overview of ongoing projects and of ongoing research projects involving personal data under the GDPR. The 2018-DCRC-0044 replaces the former notification from the Danish Data Protection Agency.

The SALiCCS research programme is conducted in compliance with the requirements of the GDPR and other applicable laws in the respective countries, as well as the respective procedures at Statistics Denmark, the Danish Cancer Society Research Center, Karolinska Institutet and the Finnish Cancer Registry. All data have been stored, linked and pooled and are analysed at a secure remote platform at Statistics Denmark, with controlled remote access only for individually approved SALiCCS project members. Personal identification numbers were replaced by pseudonymised ID numbers, and the key code is kept only by the original register holders or at the respective statistical institutes. All the results of the statistical analyses will only be presented as aggregated data.

## Specific Characteristics of the Study Population

The core SALiCCS population comprises 21,292 five-year survivors, 103,303 population comparisons and 29,644 siblings as the second comparison group. The distribution of diagnostic characteristics differed only slightly in Denmark, Finland and Sweden (**Table 2**). The most common diagnoses were tumours of the central nervous system (23%), leukaemias (23%) and lymphomas (15%). Slightly over half of the childhood cancer survivors were male (53%), 31% were diagnosed before the age of 5 years, and about 60% were diagnosed between 1990 and 2009. The length of follow-up after the date of cancer diagnosis ranged from 5.0 to 46.6 years, with a median follow-up time of 20.7 years in Denmark, 19.1 years in Finland and 20.6 years in Sweden (**Table 2**).

**Table 2:** Characteristics of 5-year childhood cancer survivors diagnosed in 1971-2008 (DEN), 2009 (FIN), 2011 (SWE) by country and for the three countries combined.

	Denmark		Finland		Sweden		Three-Country Wide pooled data	
	*N*	*%*	*N*	*%*	*N*	*%*	*N*	*%*
**Total**	5343		5672		10277		21292	
**Sex**								
Boys	3000	56.1	2939	51.8	5405	52.6	11344	53.3
Girls	2343	43.9	2733	48.2	4872	47.4	9948	46.7
**Age at diagnosis (years)**								
<1	306	5.7	377	6.7	607	5.9	1290	6.1
1 – 4	1294	24.2	1434	25.3	2475	24.1	5203	24.4
5 – 9	964	18.0	948	16.7	1844	17.9	3756	17.6
10 – 14	1046	19.6	1106	19.5	2005	19.5	4157	19.5
15 – 19	1733	32.4	1807	31.9	3346	32.6	6886	32.3
**Decade of diagnosis**								
1971 – 1979	824	15.4	797	14.1	1578	15.4	3199	15.0
1980 – 1989	1259	23.6	1316	23.2	2408	23.4	4983	23.4
1990 – 1999	1592	29.8	1765	31.1	2841	27.6	6198	29.1
2000 – 2009	1668	31.2	1794	31.6	2803	27.3	6265	29.4
2010 - 2011	–	–	–	–	647	6.3	647	3.0
**Decade of birth**								
1951 – 1959	182	3.4	235	4.1	440	4.3	857	4.0
1960 – 1969	722	13.5	645	11.4	1285	12.5	2652	12.5
1970 – 1979	1342	25.1	1310	23.1	2377	23.1	5029	23.6
1980 – 1989	1508	28.2	1728	30.5	2659	25.9	5895	27.7
1990 – 1999	1178	22.1	1270	22.4	2427	23.6	4875	22.9
2000 – 2009	411	7.7	484	8.5	1048	10.2	1943	9.1
2010 – 2011	–	–	–	–	41	0.4	41	0.2
**Cancer type****^a^**								
Leukaemias	1189	22.3	1382	24.4	2324	22.6	4895	23.0
*Lymphoid leukaemia^b^*	1000	18.7	1170	20.6	1895	18.4	4065	19.1
*Acute myeloid leukaemia^c^*	134	2.5	130	2.3	235	2.3	499	2.3
*Other leukaemia*	55	1.0	82	1.5	194	1.9	331	1.6
Lymphomas	759	14.2	952	16.8	1465	14.3	3176	14.9
*Hodgkin lymphoma^d^*	440	8.2	569	10.0	813	7.9	1822	8.6
*Non-Hodgkin lymphoma^e^*	196	3.7	364	6.4	213	2.1	773	3.6
*Other lymphoma*	123	2.3	19	0.3	439	4.3	581	2.7
CNS tumours^f^	1277	23.9	1110	19.6	2524	24.6	4911	23.1
*Ependymoma*	103	1.9	110	1.9	230	2.2	443	2.1
*Astrocytoma and other gliomas*	510	10.0	697	12.3	1088	10.6	2295	10.8
*Embryonal CNS tumours*	130	2.4	108	1.9	384	3.7	622	2.9
*Other specified or unspecified CNS tumour*	534	10.0	195	3.4	822	8.0	1551	7.3
Sympathetic nervous system tumours	169	3.2	209	3.7	244	2.4	622	2.9
Retinoblastomas	148	2.8	124	2.2	226	2.2	498	2.3
Renal tumours	232	4.3	273	4.8	487	4.7	992	4.7
Hepatic tumours	35	0.7	35	0.6	80	0.8	150	0.7
Malignant bone tumours	180	3.4	202	3.6	396	3.9	778	3.7
Soft tissue sarcomas	276	5.2	315	5.6	488	4.8	1079	5.1
Germ cell tumours	466	8.7	322	5.7	642	6.3	1430	6.7
Malignant epithelial neoplasms	533	10.0	676	11.9	1110	10.8	2319	10.9
Other & unspecified malignant neoplasms	79	1.5	72	1.3	291	2.8	442	2.1
**Secondary malignancy before age of 20 years**								
Yes	66	1.2	65	1.2	101	1.0	232	1.1
No	5277	98.8	5607	98.8	10176	99.0	21060	98.9
**End event**								
Death	569	10.7	542	9.6	831	8.1	1942	9.1
Emigration (1^st^ emigration)	391	7.3	59	1.0	433	4.2	883	4.2
Lost to follow-up	5	0.1	0	0	0	0	5	<0.1
End of study DEN: 11 Aug 2017 SWE: 31 Dec 2016 FIN: 31 Dec 2014	4378	81.9	5071	89.4	9013	87.7	18462	86.7
**Median time of follow-up in years (range)**	20.7 (5.0-46.6)		19.1 (5.0-44.0)		20.6 (5.0-46.0)		20.1 (5.0-46.6)	

No missing information for any of the characteristics given in this table.

a Classified by the International Classification of Childhood Cancer.

b Lymphoid leukaemia defined as ICCC1 group I a-b and ICCC3 group Ia.

c Acute myeloid leukaemia defined as ICCC1 group Ic and ICCC3 group Ib.

d Hodgkin lymphoma defined as ICCC1 and ICCC3 group IIa.

e Non-Hodgkin lymphoma defined as ICCC1 and ICCC3 group IIb.

f CNS tumor subtypes were grouped as follows: Ependymoma (defined by ICCC 1 and ICCC3 group 3a), astrocytoma and other gliomas (ICCC 1 and ICCC 3 groups 3b and 3d combined), and embryonal CNS tumours (defined by ICCC 1 and ICCC3 group 3c).

**Figure 2:**
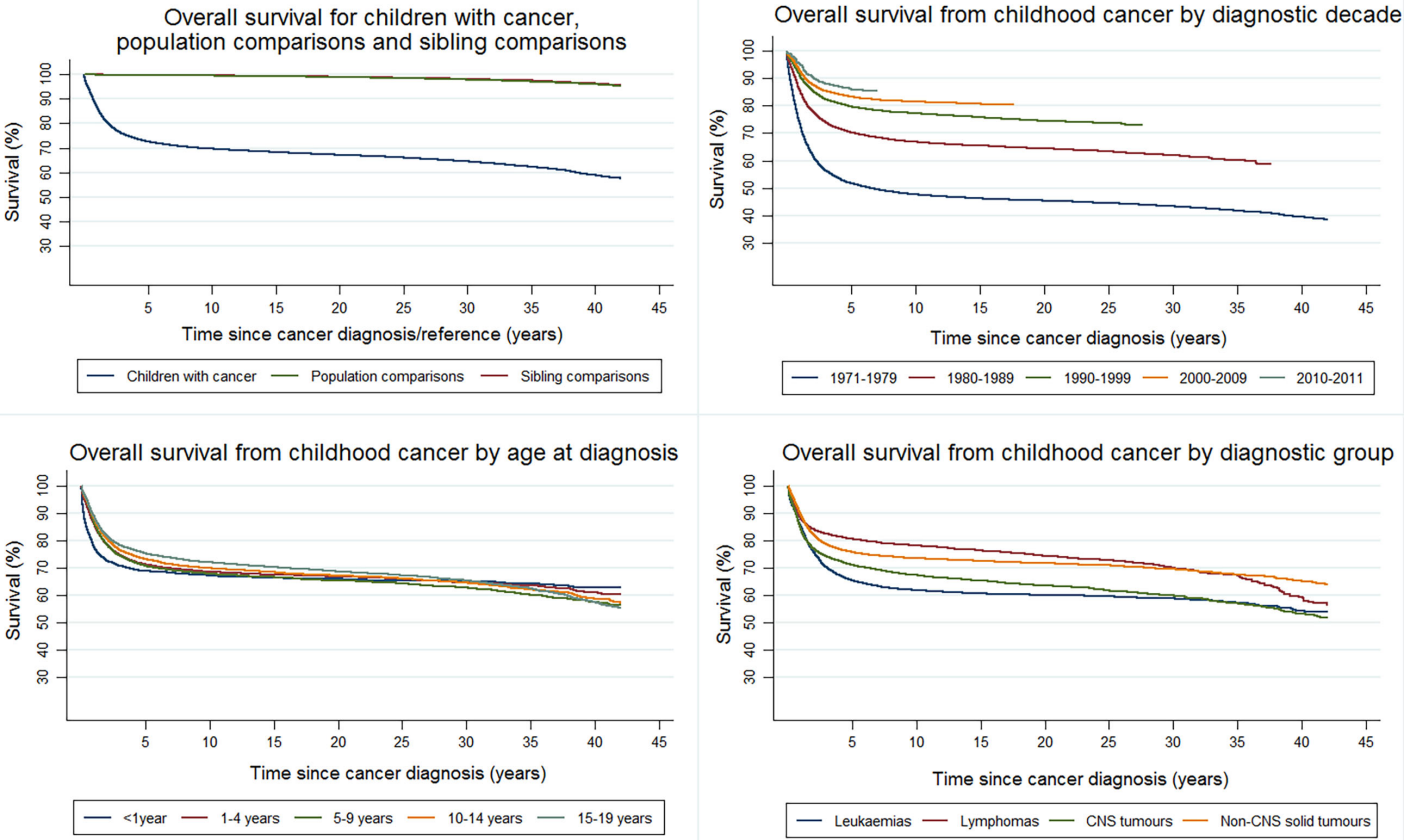
Overall survival in the SALiCCS population: by children with cancer, population comparisons and siblings, by diagnostic decade, by age at diagnosis and by diagnostic group.

The SALiCCS core population is defined by individuals who survived the five-year survival point. To describe this population, the survival probabilities of all children diagnosed with cancer in Denmark, Finland and Sweden, their population comparisons and siblings are shown in **Figure 2** (including children who died or emigrated within the first five years of diagnosis/reference date) and a comparison of the distribution of diagnostic groups and specific cancer types between all incident childhood cancer cases in Denmark, Finland and Sweden and the SALiCCS 5-year childhood cancer survivors is given in **Table S1**. Overall survival figures for this extended population are also given by diagnostic decade, age at diagnosis, diagnostic group and country (**Figure 2** and **Supplementary Material S2**–**S5**). We intentionally excluded any adverse social and socioeconomic conditions that may have arisen while being hospitalised and receiving cancer treatment by starting follow-up at five years after the childhood cancer diagnosis, since these acute and direct outcomes would not reflect the social and socioeconomic consequences in long-term survivors.

**Tables 3** and **S2** present the distribution of socio-demographic characteristics in the SALiCCS core population, consisting of all individuals who survived five years after diagnosis or the respective reference date for comparisons. The distribution of place of residence, parental age and parental socioeconomic characteristics differed only slightly for childhood cancer survivors and population and sibling comparisons (**Table 3**).

**Table 3:** Sociodemographic and socioeconomic characteristics of 5-year childhood cancer survivors, population comparisons and siblings, pooled data from the three Nordic countries.

	Three-country wide pooled data					
	Childhood cancer survivors		Population comparisons		Siblings	
	N	%	N	%	N	%
**Total**	21292		103303		29644	
**Sex**						
Boys	11334	53.3	55134	53.4	15137	51.1
Girls	9948	46.7	48169	46.6	14507	48.9
**Decade of birth**						
1951 – 1959	857	4.0	4129	4.0	1065	3.6
1960 – 1969	2652	12.5	12884	12.5	4166	14.1
1970 – 1979	5029	23.6	24381	23.6	6908	23.3
1980 – 1989	5895	27.7	28516	27.6	8650	29.2
1990 – 1999	4875	22.9	23738	23.0	6667	22.5
2000 – 2009	1943	9.1	9454	9.2	2149	7.3
2010 – 2011	41	0.2	201	0.2	39	0.1
**Region of residence^a,b^**						
Major city	6363	32.4	30699	32.1	8301	30.2
Town & suburb	7354	37.4	35606	37.3	10085	36.7
Rural areas	5743	29.2	28086	29.4	8652	31.5
Missing^a^	211	1.1	1177	1.2	453	1.7
**Parents**						
Biological mothers	21073	99.0	101873	98.6	29447	99.3
Biological fathers	20763	97.5	100179	97.0	29159	98.4
**Maternal age** ^c,d^						
≤25	7684	36.1	38420	37.2	11853	40.0
26 – 30	7297	34.3	34561	33.5	10086	34.0
31 – 35	4299	20.2	20484	19.8	5585	18.8
36 – 40	1535	7.2	7175	7.0	1705	5.8
41 – 45	250	1.2	1198	1.2	214	0.7
≥46	11	0.1	54	0.1	4	<0.1
Missing	216	1.0	1411	1.4	197	0.7
**Paternal age** ^c,d^						
≤25	4338	20.4	21644	21.0	6599	22.3
26 – 30	7034	33.0	33657	32.6	9960	33.6
31 – 35	5411	25.4	26278	25.4	7633	25.8
36 – 40	2647	12.4	12394	12.0	3422	11.5
41 – 45	944	4.4	4404	4.3	1124	3.8
≥46	409	1.9	1981	1.9	419	1.4
Missing	509	2.4	2945	2.9	487	1.6
**Maternal education** ^a,c,d,e^						
Short	5729	28.1	29109	29.4	8821	31.2
Medium	7979	39.1	37964	38.4	10563	37.3
Higher	5249	25.7	24251	24.5	6702	23.7
Missing^a^	1437	7.1	7614	7.7	2231	7.9
**Paternal education** ^a,c,d,e^						
Short	5643	27.7	28015	28.3	8690	30.7
Medium	8514	41.8	40679	41.1	11269	39.8
Higher	4712	23.1	21868	22.1	6213	21.9
Missing^a^	1525	7.5	8376	8.5	2145	7.6
**Maternal employment status** ^a,c,d^						
Employed	14447	74.2	69027	73.0	18293	67.4
Unemployed	4760	24.5	23841	25.2	7976	29.4
Missing^a^	257	1.3	1703	1.8	860	3.2
**Paternal employment status** ^a,c,d^						
Employed	16673	85.7	80048	84.6	22666	85.6
Unemployed	2269	11.7	11296	12.0	3339	12.3
Missing^a^	522	2.7	3227	3.4	1124	4.1
**Maternal disposable income** ^a,c,d,f^						
1^st^ quartile	3791	22.0	18680	22.4	5207	22.1
2^nd^ quartile	4044	23.5	20138	24.2	5269	22.4
3^rd^ quartile	4508	26.2	21446	25.7	5839	24.8
4^th^ quartile	4591	26.7	21401	25.7	6582	27.9
Missing^a^	266	1.6	1686	2.0	678	2.9
**Paternal disposable income** ^a,c,d,f^						
1^st^ quartile	3732	21.7	18892	22.7	5353	22.7
2^nd^ quartile	4080	23.7	20485	24.6	5651	24.0
3^rd^ quartile	4419	25.7	20776	24.9	5824	24.7
4^th^ quartile	4458	25.9	20149	24.2	5861	24.9
Missing^a^	511	3.0	3049	3.7	886	3.8
**Average days/year with hospital visit** ^g^						
	Average days/year		Average days/year		Average days/year	
0-4 years after reference date	18.2		0.8		0.9	
5-9 years after reference date	6.2		1.0		1.1	
10-14 years after reference date	3.8		1.3		1.4	
15-19 years after reference date	3.4		1.4		1.6	
20-24 years after reference date	3.4		1.6		1.8	
25-29 years after reference date	3.9		1.8		1.9	
30-34 years after reference date	4.4		1.7		1.9	
35-39 years after reference date	5.5		1.8		2.4	
**End event**						
Death	1942	9.1	1416	1.4	389	1.3
Emigration (1^st^ emigration)	883	4.2	5606	5.4	1526	5.2
Lost to follow-up	5	<0.1	26	<0.1	3	<0.1
End of study DEN: 11 Aug 2017 SWE: 31 Dec 2016 FIN: 31 Dec 2014	18462	86.7	96255	93.2	27726	93.5
**Median time of follow-up (years)**	20.1 (5.0-46.6)		21.4 (5.0-46.6)		22.1 (5.0-46.6)	
**Type of sibling** ^h^						
Full sibling	–	–	–	–	24710	83.4
Half sibling	–	–	–	–	4934	16.6
**Age difference between survivor and sibling**						
Less than 5 years older	–	–	–	–	9827	33.2
5-10 years older	–	–	–	–	6009	20.3
Less than 5 years younger	–	–	–	–	8807	29.7
5-10 years younger	–	–	–	–	5001	16.9

Reference date corresponds to the date of diagnosis for the population comparisons. For siblings the reference date corresponds to the date when a sibling was of the same age as the respective survivor at diagnosis.

a Information tied to the years of register coverage in the respective country (see **Table 1**). Parental socioeconomic information from Finland were tied to the years from 1980 onwards.

b Corresponds to reference year.

c Characteristics correspond to the biological parents.

d Corresponds to the year before reference year; if not available, then to the year closest to the year before reference year.

e Parental highest education was grouped into short [early childhood education, primary and lower secondary education, ISCED levels 0-2], medium [upper secondary including vocational upper secondary education, ISCED level 3] and higher [ISCED level 4-8] education, following the International Standard Classification of Education (ISCED). Parents with missing education in Finland have been allocated to lowest education category, as only education from secondary level and above is registered in Finland.

f Annual disposable income was categorised into four groups based on the sex- and calendar-year specific income distribution (quartiles) of the population comparisons in the respective country.

g Average number of days/year with inpatient and outpatient hospital contact for any somatic and mental disorders during 5-year periods after reference date.

h Adoptive siblings from Sweden were assigned to the group of full siblings, whereas adoptive siblings from Denmark cannot be specifically identified and therefore may be found in both groups.

This SALiCCS core population will serve as the basis for individual studies of the objectives of the SALiCCS research programme, as outlined in section 1.3. Additional inclusion and exclusion criteria may be applied to the core population in individual SALiCCS studies, which may be restricted to specific time periods, depending on the respective research objectives and register coverage.

## Discussion

The growing number of survivors of childhood cancers diagnosed at a young age, with many years of life ahead, indicates the increasing clinical and public health relevance of investigating the long-term social and socioeconomic consequences of the cancer diagnosis and the life-saving treatment. Some previous evidence points to higher risks of impaired social functioning and adverse socioeconomic outcomes in adult life. Nevertheless, additional research is urgently required to fully understand the long-term social consequences of a diagnosis of cancer in childhood, to identify vulnerable survivors at particular risk for adverse social and socioeconomic impairments in adulthood and ultimately to provide scientific knowledge for evidenced-based survivorship care that addresses not only somatic late effects but also psychosocial impairments.

Data linkage among various population-based registries in Denmark, Finland and Sweden gave us the unique possibility of setting up the largest, most comprehensive population-based research initiative on the social and socioeconomic consequences of childhood cancer so far, comprising more than 21,000 five-year childhood cancer survivors and two independent comparison groups. The large number of survivors will enable detailed analyses, allowing for identification of vulnerable subgroups in terms of e.g. diagnostic or socio-demographic characteristics. Use of high-quality population-based register data with high coverage, virtually no loss to follow-up and no self-reporting or non-participation allows reliable estimation of the social and socioeconomic outcomes with minimal risk of bias in countries with similar welfare systems and generally equal access to education and other services. Particularly valuable strengths of the SALiCCS research programme include annual information on social and socioeconomic outcomes, facilitating the study of trajectories and enabling a comprehensive long-term follow-up, as well as the availability of detailed information on hospital contacts, allowing stratified analyses by somatic or mental late effects. A limitation of the SALiCCS research programme is the lack of comprehensive and three-countrywide information on cancer treatment and other relevant clinical characteristics such as subtype of disease and tumour stage or grade. Such information would have been of considerable value for assessing the underlying mechanisms leading to adverse social and socioeconomic conditions, and to identify survivors of childhood cancer that are particularly vulnerable to adverse outcomes. For instance, especially cranial radiation therapy has been associated with various somatic late effects, including long-term neurocognitive impairment, as well as with adverse educational attainments, higher risk of unemployment and low income (22). Understanding the causal pathway of those adverse outcomes would be of substantial clinical and public health relevance. We do, however, have information on hospital contacts, including the full history, with dates of in- and outpatient hospital contacts including corresponding diagnoses, which may be used in some individual SALiCCS studies as indicators of severity of disease, length of treatment and disease burden.

The novel findings resulting from this research programme may serve as a basis for recommendations on interventions for vulnerable subgroups of survivors. Such recommendations may not be limited to the Nordic countries, with their extensive welfare systems, but may also be applicable to other countries, especially within Europe.

## Data Availability Statement

The data that support the information of this article were accessed remotely on a secure platform at Statistics Denmark. Pseudonymised individual-level data were obtained from national registry holders after ethical approval (where applicable) and secrecy assessment. According to Danish, Finnish and Swedish laws and regulations, individual-level sensitive data can only be made available for researchers who fulfil legal requirements for access to personal sensitive data. Please contact Jeanette Falck Winther (jeanette@cancer.dk), the Principal Investigator of the SALiCCS research programme, for further questions about data access.

## Ethics Statement

The SALiCCS research programme has been approved by Statistics Denmark, the Regional Ethical Review Board in Stockholm, Sweden (dnr 2016/25-31/5, 2016/1561-32, 2017/1656-32, 2017/1990-32, 2017/2340-32, 2018/1165-32), Findata (Dnro THL/5543/14.06.00/2020) prolonging the former approvals by the National Institute for Health and Welfare and Social Insurance (KELA) and Statistics Finland (TK-53-394-17) in Finland. For the European Union General Data Protection Regulation (GDPR), the SALiCCS project is listed in a local archive (2018-DCRC-0044) at the Danish Cancer Society Research Center, which provides an accurate, updated overview of ongoing projects and of ongoing research projects involving personal data under the GDPR. The 2018-DCRC-0044 replaces the former notification from the Danish Data Protection Agency.

## Author Contributions

FE and JW developed the concept and outline of the manuscript. All authors contributed to the acquisition and preparation of data. FE, LF, HM, NM, L-MM-H, MF, and JW developed the strategy for the descriptive analysis and presentation of the study population. FE, LF, HM, and JW drafted the manuscript. All authors provided critical feedback, critically reviewed the manuscript for important intellectual content, and revised the manuscript. All authors approved the final manuscript as submitted and agreed to be accountable for all aspects of the work.

## Funding

This work was supported by NordForsk under grant 76111, the Danish Childhood Cancer Foundation under grant 2016-0293, Aarhus University under fellowship 43239402, the Swiss National Science Foundation under grant P2LUP3_175288 (personal fellowship to LM), the Tømrermester Jørgen Holm og Hustru Elisa F. Hansens Mindelegat under grant 20088 and the Swedish Childhood Cancer Foundation under grant PR2020-0130. The funding sources had no involvement in the content or preparation of the manuscript.

## Conflict of Interest

The authors declare that the research was conducted in the absence of any commercial or financial relationships that could be construed as a potential conflict of interest.

## Publisher’s Note

All claims expressed in this article are solely those of the authors and do not necessarily represent those of their affiliated organizations, or those of the publisher, the editors and the reviewers. Any product that may be evaluated in this article, or claim that may be made by its manufacturer, is not guaranteed or endorsed by the publisher.

## Supplementary Material

Supplementary Figure 1 Flow diagram of the sampling of the SALiCCS core population.

Supplementary Figure 2 Overall survival for children with cancer, population comparisons and sibling, for the entire SALiCCS population and by country (reference date in 1971-2008 (DEN), 2009 (FIN), 2011 (SWE)).

Supplementary Figure 3 Overall survival from childhood cancer by diagnostic decade, for the entire SALiCCS population and by country (children diagnosed with cancer in 1971-2008 (DEN), 2009 (FIN), 2011 (SWE)).

Supplementary Figure 4 Overall survival from childhood cancer by age at diagnosis, for the entire SALiCCS population and by country (children diagnosed with cancer in 1971-2008 (DEN), 2009 (FIN), 2011 (SWE)).

Supplementary Figure 5 Overall survival from childhood cancer by diagnostic group, for the entire SALiCCS population and by country (children diagnosed with cancer in 1971-2008 (DEN), 2009 (FIN), 2011 (SWE)).

Supplementary Figure 6 Overall survival from childhood non-CNS solid tumours by tumour type, for the entire SALiCCS population and by country (children diagnosed with cancer in 1971-2008 (DEN), 2009 (FIN), 2011 (SWE)).

Supplementary Table 1 Comparison of the distribution of diagnostic groups and specific cancer types between all incident childhood cancer cases and the SALiCCS 5-year childhood cancer survivors.

Supplementary Table 2 Sociodemographic and socioeconomic characteristics of 5-year childhood cancer survivors, population comparisons and siblings by country.

## Abbreviations

ALiCCS, Adult Life after Childhood Cancer in Scandinavia; CNS, Central Nervous System; NOPHO, Nordic Society of Paediatric Haematology and Oncology; SALiCCS, Socioeconomic Consequences in Adult Life after Childhood Cancer in Scandinavia.
