# Supplementary figures and images for: Cohort Profile: The Socioeconomic Consequences in Adult Life After Childhood Cancer in Scandinavia (SALiCCS) Research Programme
Source: Front Oncol. 2021 Nov 26;11:752948. doi: 10.3389/fonc.2021.752948 (PMC8662544; doi:10.3389/fonc.2021.752948)

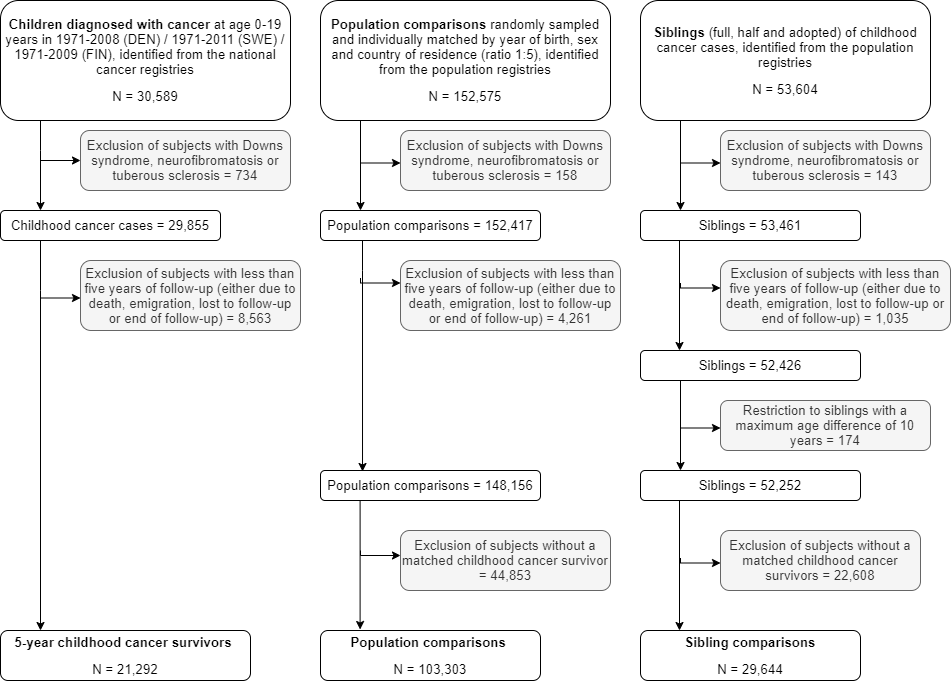

Supplement: Supplementary Figure 1 — Flow diagram of the sampling of the SALiCCS core population. [file Image_1.jpeg]

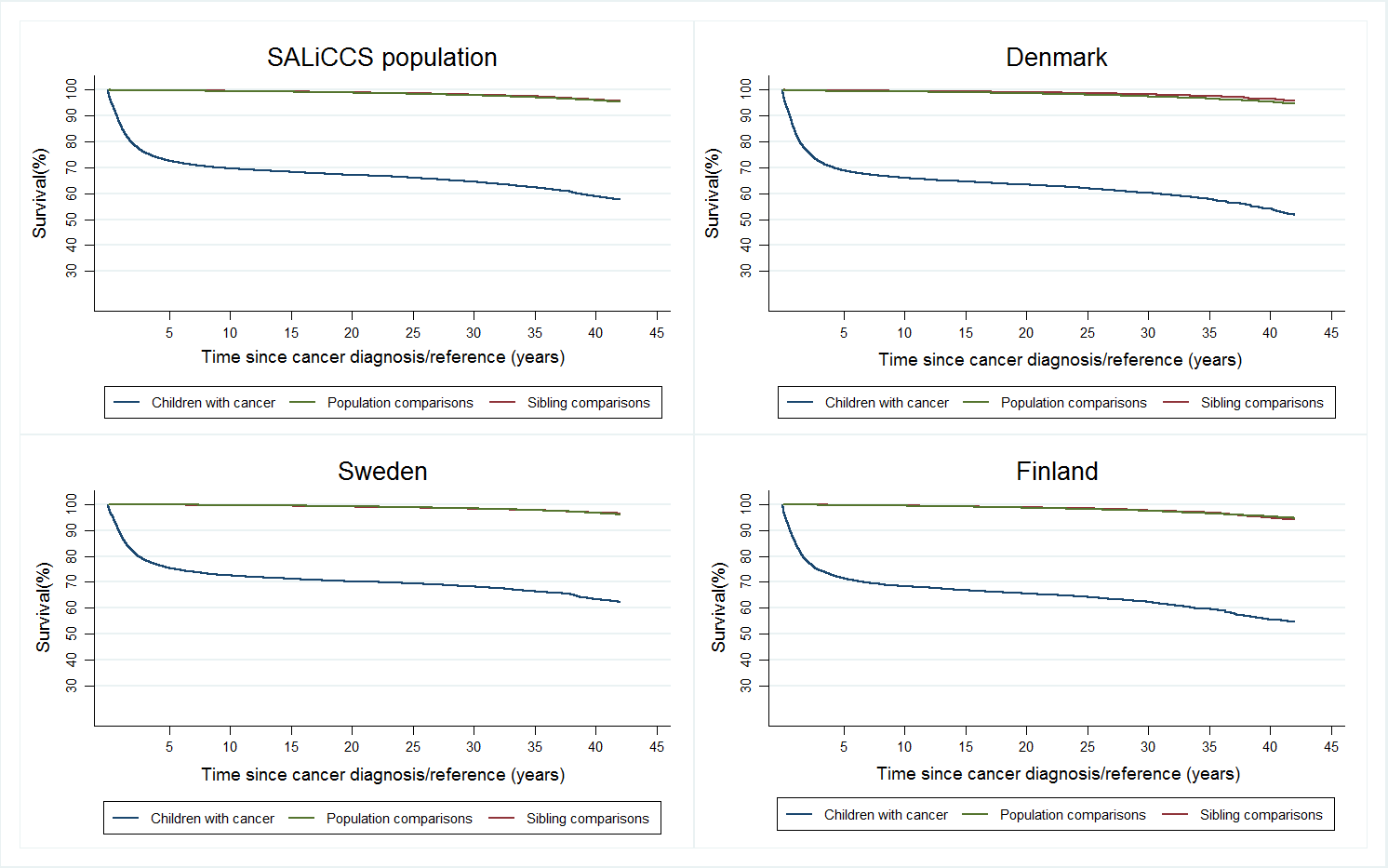

Supplement: Supplementary Figure 2 — Overall survival for children with cancer, population comparisons and sibling, for the entire SALiCCS population and by country (reference date in 1971-2008 (DEN), 2009 (FIN), 2011 (SWE)). [file Image_2.jpeg]

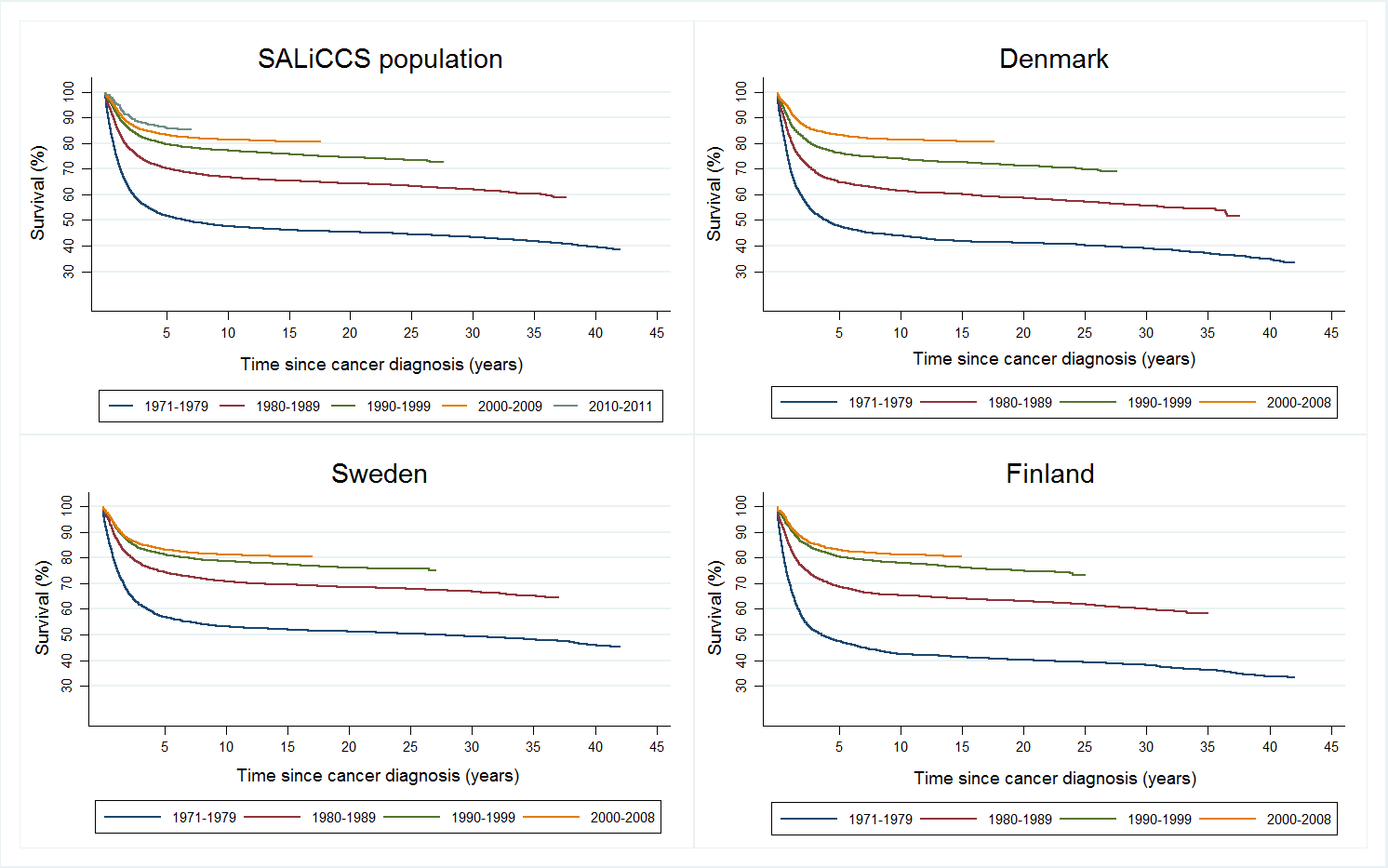

Supplement: Supplementary Figure 3 — Overall survival from childhood cancer by diagnostic decade, for the entire SALiCCS population and by country (children diagnosed with cancer in 1971-2008 (DEN), 2009 (FIN), 2011 (SWE)). [file Image_3.jpeg]

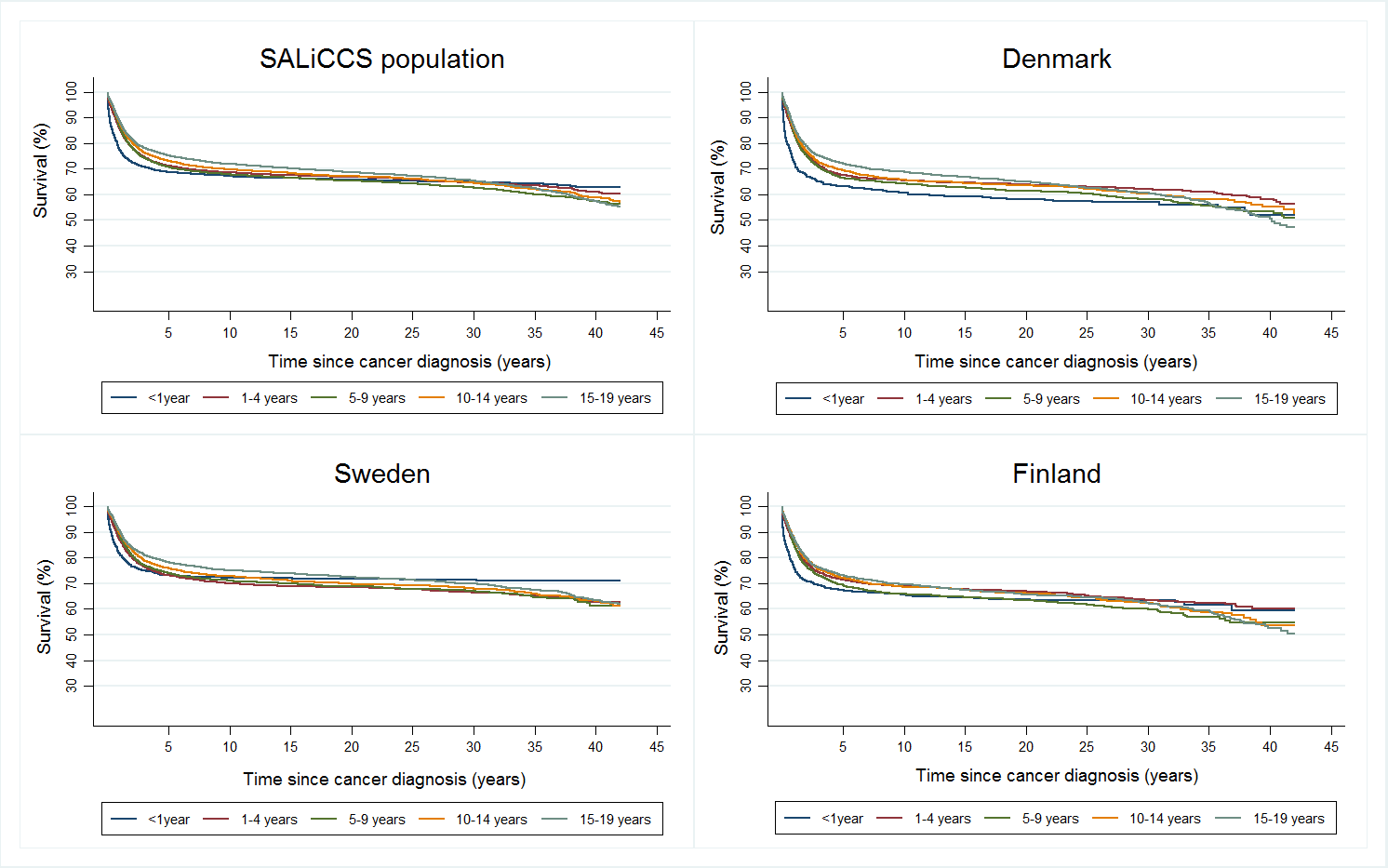

Supplement: Supplementary Figure 4 — Overall survival from childhood cancer by age at diagnosis, for the entire SALiCCS population and by country (children diagnosed with cancer in 1971-2008 (DEN), 2009 (FIN), 2011 (SWE)). [file Image_4.jpeg]

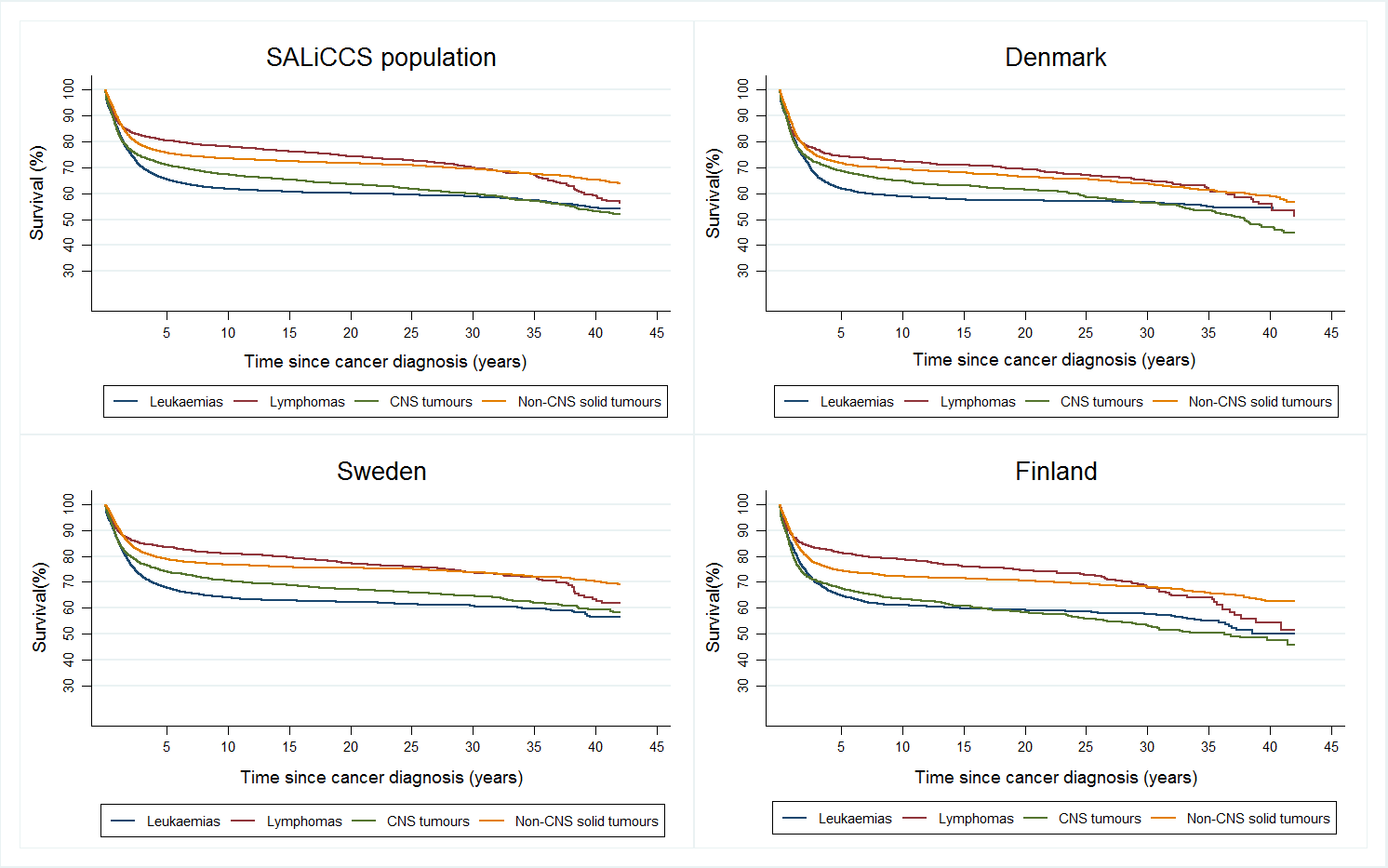

Supplement: Supplementary Figure 5 — Overall survival from childhood cancer by diagnostic group, for the entire SALiCCS population and by country (children diagnosed with cancer in 1971-2008 (DEN), 2009 (FIN), 2011 (SWE)). [file Image_5.jpeg]

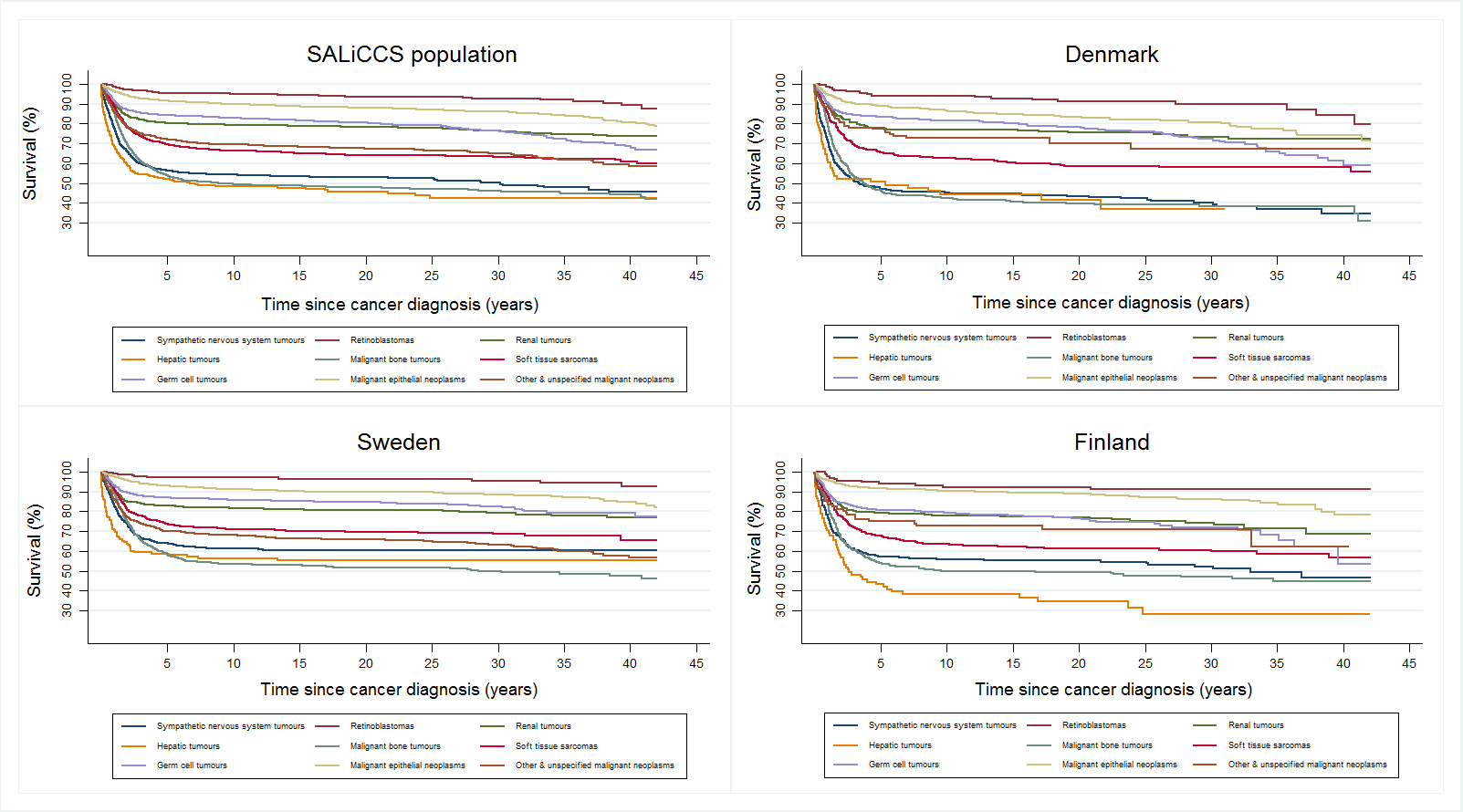

Supplement: Supplementary Figure 6 — Overall survival from childhood non-CNS solid tumours by tumour type, for the entire SALiCCS population and by country (children diagnosed with cancer in 1971-2008 (DEN), 2009 (FIN), 2011 (SWE)). [file Image_6.jpeg]
