# Supplementary material for: Cohort Profile: The Socioeconomic Consequences in Adult Life After Childhood Cancer in Scandinavia (SALiCCS) Research Programme
Source: Front Oncol. 2021 Nov 26;11:752948. doi: 10.3389/fonc.2021.752948 (PMC8662544; doi:10.3389/fonc.2021.752948)
Supplement: Supplementary Table 1 — Comparison of the distribution of diagnostic groups and specific cancer types between all incident childhood cancer cases and the SALiCCS 5-year childhood cancer survivors. [file Table_1.docx]

**^Supplementary tables to the article:^**

**^Cohort Profile: The Socioeconomic Consequences in Adult Life after Childhood Cancer in Scandinavia (SALiCCS) Research Programme^**

| **TABLE S1: COMPARISON OF THE DISTRIBUTION OF DIAGNOSTIC GROUPS AND SPECIFIC CANCER TYPES BETWEEN ALL INCIDENT CHILDHOOD CANCER CASES AND THE SALICCS 5-YEAR CHILDHOOD CANCER SURVIVORS^A^** | | | | |
| --- | --- | --- | --- | --- |
|  | **ALL CHILDHOOD CANCER CASES** | | **5-YEAR SURVIVORS** | |
|  | ***N*** | ***%*** | ***N*** | ***%*** |
| **Total** | 29855 |  | 21292 |  |
| **Leukaemias** | 7620 | 25.5 | 4895 | 23.0 |
| Lymphoid leukaemia^b^ | 5660 | 19.0 | 4065 | 19.1 |
| Acute myeloid leukaemia^c^ | 1168 | 3.9 | 499 | 2.3 |
| Other leukaemia | 792 | 2.7 | 331 | 1.6 |
| **Lymphomas** | 3997 | 13.4 | 3176 | 14.9 |
| Hodgkin lymphoma^d^ | 2006 | 6.7 | 1822 | 8.6 |
| Non-Hodgkin lymphoma^e^ | 1120 | 3.8 | 773 | 3.6 |
| Other lymphoma | 871 | 2.9 | 581 | 2.7 |
| **CNS tumours^f^** | 7068 | 23.7 | 4911 | 23.1 |
| Ependymoma | 685 | 2.3 | 443 | 2.1 |
| Astrocytoma and other gliomas | 3136 | 10.5 | 2295 | 10.8 |
| Embryonal CNS tumours | 1224 | 4.1 | 622 | 2.9 |
| Other specified or unspecified CNS tumour | 2023 | 6.8 | 1551 | 7.3 |
| **Sympathetic nervous system tumours** | 1133 | 3.8 | 622 | 2.9 |
| **Retinoblastomas** | 526 | 1.8 | 498 | 2.3 |
| **Renal tumours** | 1255 | 4.2 | 992 | 4.7 |
| **Hepatic tumours** | 300 | 1.0 | 150 | 0.7 |
| **Malignant bone tumours** | 1460 | 4.9 | 778 | 3.7 |
| **Soft tissue sarcomas** | 1573 | 5.3 | 1079 | 5.1 |
| **Germ cell tumours** | 1719 | 5.8 | 1430 | 6.7 |
| **Malignant epithelial neoplasms** | 2575 | 8.6 | 2319 | 10.9 |
| **Other & unspecified malignant neoplasms** | 629 | 2.1 | 442 | 2.1 |

^a^Diagnosed in 1971-2008 (DEN), 2009 (FIN), 2011 (SWE).

^b^Lymphoid leukaemia defined as ICCC1 group I a-b and ICCC3 group Ia.

^c^Acute myeloid leukaemia defined as ICCC1 group Ic and ICCC3 group Ib.

^d^Hodgkin lymphoma defined as ICCC1 and ICCC3 group IIa.

^e^Non-Hodgkin lymphoma defined as ICCC1 and ICCC3 group IIb.

^f^CNS tumor subtypes were grouped as follows: Ependymoma (defined by ICCC 1 and ICCC3 group 3a), astrocytoma and other gliomas (ICCC 1 and ICCC 3 groups 3b and 3d combined), and embryonal CNS tumours (defined by ICCC 1 and ICCC3 group 3c).

| **Table S2: Sociodemographic and socioeconomic Characteristics of 5-year childhood cancer survivors, population comparisons and siblings by country** | | | | | | | | | | | | | | | | | | |
| --- | --- | --- | --- | --- | --- | --- | --- | --- | --- | --- | --- | --- | --- | --- | --- | --- | --- | --- |
|  | **DENmark** | | | | | | **finland** | | | | | | **Sweden** | | | | | |
|  | **survivors** | | **population comparisons** | | **Siblings** | | **survivors** | | **population comparisons** | | **siblings** | | **survivors** | | **population comparisons** | | **siblings** | |
|  | N | % | N | % | N | % | N | % | N | % | N | % | N | % | N | % | N | % |
| **Total** | 5343 |  | 25033 |  | 6723 |  | 5672 |  | 28005 |  | 8399 |  | 10277 |  | 50265 |  | 14522 |  |
| **Sex** | | | | | | | | | | | | | | | | | | |
| Boys | 3000 | 56.2 | 14122 | 56.4 | 3438 | 51.1 | 2939 | 51.8 | 14525 | 51.9 | 4232 | 50.4 | 5405 | 52.6 | 26487 | 52.7 | 7467 | 51.4 |
| Girls | 2343 | 43.8 | 10911 | 43.6 | 3285 | 48.9 | 2733 | 48.2 | 13480 | 48.1 | 4167 | 49.6 | 4872 | 47.4 | 23778 | 47.3 | 7055 | 48.6 |
| **Decade of birth** | | | | | | | | | | | | | | | | | | |
| 1951 – 1959 | 182 | 3.4 | 859 | 3.4 | 217 | 3.2 | 235 | 4.1 | 1116 | 4.0 | 344 | 4.1 | 440 | 4.3 | 2154 | 4.3 | 504 | 3.5 |
| 1960 – 1969 | 722 | 13.5 | 3398 | 13.6 | 1073 | 16.0 | 645 | 11.4 | 3170 | 11.3 | 1073 | 12.8 | 1285 | 12.5 | 6316 | 12.6 | 2020 | 13.9 |
| 1970 – 1979 | 1342 | 25.1 | 6262 | 25.0 | 1744 | 25.9 | 1310 | 23.1 | 6482 | 23.2 | 1875 | 22.3 | 2377 | 23.1 | 11637 | 23.2 | 3289 | 22.7 |
| 1980 – 1989 | 1508 | 28.2 | 6931 | 27.7 | 1842 | 27.4 | 1728 | 30.5 | 8560 | 30.6 | 2569 | 30.6 | 2659 | 25.9 | 13025 | 25.9 | 4239 | 29.2 |
| 1990 – 1999 | 1178 | 22.1 | 5623 | 22.5 | 1426 | 21.2 | 1270 | 22.4 | 6283 | 22.4 | 1927 | 22.9 | 2427 | 23.6 | 11832 | 23.5 | 3314 | 22.8 |
| 2000 – 2009 | 411 | 7.7 | 1960 | 7.8 | 421 | 6.3 | 484 | 8.5 | 2394 | 8.9 | 611 | 7.3 | 1048 | 10.2 | 5100 | 10.2 | 1117 | 7.7 |
| 2010 – 2011 | - | - | - | - |  |  | - | - |  |  |  |  | 41 | 0.4 | 201 | 0.4 | 39 | 0.3 |
| **Region of residence**^a,b^ | | | | | | | | | | | | | | | | | | |
| Major city | 1235 | 27.3 | 5752 | 27.2 | 1428 | 25.0 | 1479 | 30.3 | 7250 | 30.0 | 1957 | 26.9 | 3649 | 35.5 | 17697 | 35.2 | 4916 | 33.9 |
| Town & suburb | 1490 | 33.0 | 6887 | 32.6 | 1838 | 32.2 | 1509 | 31.0 | 7377 | 30.6 | 2143 | 29.5 | 4355 | 42.4 | 21342 | 42.5 | 6104 | 42.0 |
| Rural areas | 1609 | 35.6 | 7407 | 35.0 | 2165 | 38.0 | 1861 | 38.2 | 9453 | 39.2 | 3074 | 42.3 | 2273 | 22.1 | 11226 | 22.3 | 3413 | 23.5 |
| Missing^a^ | 185 | 4.1 | 1115 | 5.3 | 274 | 4.8 | 26 | 0.5 | 62 | 0.3 | 90 | 1.2 | 0 | 0 | 0 | 0 | 89 | 0.6 |
| **Parents** | | | | | | | | | | | | | | | | | | |
| Biological mothers | 5248 | 98.2 | 24266 | 96.9 | 6661 | 99.1 | 5658 | 99.8 | 27895 | 99.6 | 8388 | 99.9 | 10167 | 98.9 | 49712 | 98.9 | 14398 | 99.2 |
| Biological fathers | 5174 | 96.8 | 23876 | 95.4 | 6616 | 98.4 | 5545 | 97.8 | 27241 | 97.3 | 8281 | 98.6 | 10044 | 97.7 | 49062 | 97.6 | 14262 | 98.2 |
| **Maternal age**^c,d^ | | | | | | | | | | | | | | | | | | |
| ≤ 25 | 2026 | 37.9 | 9740 | 38.9 | 2912 | 43.3 | 2030 | 35.8 | 10333 | 36.9 | 3260 | 38.8 | 3628 | 35.3 | 18347 | 36.5 | 5681 | 39.1 |
| 26 – 30 | 1863 | 34.9 | 8505 | 34.0 | 2303 | 34.3 | 1913 | 33.7 | 9326 | 33.3 | 2835 | 33.8 | 3521 | 34.3 | 16730 | 33.3 | 4948 | 34.1 |
| 31 – 35 | 993 | 18.6 | 4497 | 18.0 | 1148 | 17.1 | 1192 | 21.0 | 5554 | 19.8 | 1653 | 19.7 | 2114 | 20.6 | 10433 | 20.8 | 2784 | 19.2 |
| 36 – 40 | 314 | 5.9 | 1328 | 5.3 | 266 | 4.0 | 442 | 7.8 | 2261 | 8.1 | 553 | 6.6 | 779 | 7.6 | 3586 | 7.1 | 886 | 6.1 |
| 41 – 45 | 51 | 1.0 | 180 | 0.7 | 32 | 0.5 | 81 | 1.4 | 422 | 1.5 | 84 | 1.0 | 118 | 1.2 | 596 | 1.2 | 98 | 0.7 |
| ≥46 | 1 | <0.1 | 16 | 0.1 | 0 | 0.0 | 3 | 0.1 | 18 | 0.1 | 3 | <0.1 | 7 | 0.1 | 20 | <0.1 | 1 | <0.1 |
| Missing | 95 | 1.8 | 767 | 3.1 | 62 | 0.9 | 11 | 0.2 | 91 | 0.3 | 11 | 0.1 | 110 | 1.1 | 553 | 1.1 | 124 | 0.9 |
| **Paternal age**^c,d^ | | | | | | | | | | | | | | | | | | |
| ≤ 25 | 1082 | 20.3 | 5347 | 21.4 | 1576 | 22.4 | 1251 | 22.1 | 6229 | 22.2 | 1940 | 23.1 | 2005 | 19.5 | 10068 | 20.0 | 3083 | 21.2 |
| 26 – 30 | 1905 | 35.7 | 8304 | 33.2 | 2355 | 35.0 | 1844 | 32.5 | 9122 | 32.6 | 2805 | 33.4 | 3285 | 32.0 | 16231 | 32.3 | 4800 | 33.1 |
| 31 – 35 | 1283 | 24.0 | 6155 | 24.6 | 1703 | 25.3 | 1435 | 25.3 | 7027 | 25.1 | 2150 | 25.6 | 2693 | 26.2 | 13096 | 26.1 | 3780 | 26.0 |
| 36 – 40 | 603 | 11.3 | 2751 | 11.0 | 710 | 10.6 | 698 | 12.3 | 3358 | 12.0 | 956 | 11.4 | 1346 | 13.1 | 6285 | 12.5 | 1756 | 12.1 |
| 41 – 45 | 209 | 3.9 | 889 | 3.6 | 187 | 2.8 | 246 | 4.3 | 1186 | 4.2 | 329 | 3.9 | 489 | 4.8 | 2329 | 4.6 | 608 | 4.2 |
| ≥46 | 92 | 1.7 | 430 | 1.7 | 85 | 1.3 | 91 | 1.6 | 498 | 1.8 | 99 | 1.2 | 226 | 2.2 | 1053 | 2.1 | 235 | 1.6 |
| Missing | 169 | 3.2 | 1157 | 4.6 | 107 | 1.6 | 107 | 1.9 | 585 | 2.1 | 120 | 1.4 | 233 | 2.3 | 1203 | 2.4 | 260 | 1.8 |
| **Maternal education**^a,c,d,e^ | | | | | | | | | | | | | | | | | | |
| Short | 1733 | 32.4 | 8326 | 33.3 | 2494 | 37.1 | 1230 | 25.8 | 6398 | 27.1 | 2166 | 30.6 | 2766 | 26.9 | 14385 | 28.6 | 4161 | 28.7 |
| Medium | 1875 | 35.1 | 8513 | 34.0 | 2168 | 32.3 | 1945 | 40.7 | 9500 | 40.2 | 2782 | 39.3 | 4159 | 40.5 | 19951 | 39.7 | 5613 | 38.7 |
| Higher | 1308 | 24.5 | 5621 | 22.5 | 1551 | 23.1 | 1594 | 33.4 | 7674 | 32.5 | 2119 | 30.0 | 2347 | 22.8 | 10956 | 21.8 | 3032 | 20.9 |
| Missing^a^ | 427 | 8.0 | 2573 | 10.3 | 510 | 7.6 | 5 | 0.1 | 68 | 0.3 | 5 | 0.1 | 1005 | 9.8 | 4973 | 9.9 | 1716 | 11.8 |
| **Paternal education**^a,c,d,e^ | | | | | | | | | | | | | | | | | | |
| Short | 1325 | 24.8 | 6349 | 25.4 | 1900 | 28.3 | 1384 | 29.0 | 6998 | 29.6 | 2379 | 33.6 | 2934 | 28.6 | 14668 | 29.2 | 4411 | 30.4 |
| Medium | 2351 | 44.0 | 10439 | 41.7 | 2877 | 42.8 | 1894 | 39.7 | 9291 | 39.3 | 2622 | 37.1 | 4269 | 41.5 | 20949 | 41.7 | 5770 | 39.7 |
| Higher | 1143 | 21.4 | 5180 | 20.7 | 1372 | 20.4 | 1414 | 29.6 | 6789 | 28.7 | 1996 | 28.2 | 2155 | 21.0 | 9899 | 19.7 | 2845 | 19.6 |
| Missing^a^ | 524 | 9.8 | 3065 | 12.2 | 574 | 8.5 | 82 | 1.7 | 562 | 2.4 | 75 | 1.1 | 919 | 8.9 | 4749 | 9.5 | 1496 | 10.3 |
| **Maternal employment status** ^a,c,d^ | | | | | | | | | | | | | | | | | | |
| Employed | 3433 | 77.8 | 15287 | 74.0 | 3967 | 71.7 | 3429 | 71.8 | 16869 | 71.4 | 4379 | 61.9 | 7585 | 73.8 | 36871 | 73.4 | 9947 | 68.5 |
| Unemployed | 903 | 20.5 | 4671 | 22.6 | 1451 | 26.2 | 1323 | 27.7 | 6630 | 28.1 | 2254 | 31.9 | 2534 | 24.7 | 12540 | 25.0 | 4271 | 29.4 |
| Missing^a^ | 77 | 1.7 | 708 | 3.4 | 117 | 2.1 | 22 | 0.5 | 141 | 0.6 | 439 | 6.2 | 158 | 1.5 | 854 | 1.7 | 304 | 2.1 |
| **Paternal employment status** ^a,c,d^ | | | | | | | | | | | | | | | | | | |
| Employed | 3774 | 85.5 | 17101 | 82.8 | 4689 | 84.7 | 4054 | 84.9 | 19777 | 83.7 | 5726 | 81.0 | 8845 | 86.1 | 43170 | 85.9 | 12251 | 84.4 |
| Unemployed | 503 | 11.4 | 2532 | 12.3 | 688 | 12.4 | 617 | 12.9 | 3180 | 13.5 | 807 | 11.4 | 1149 | 11.2 | 5584 | 11.1 | 1844 | 12.7 |
| Missing^a^ | 136 | 3.1 | 1033 | 5.0 | 158 | 2.9 | 103 | 2.2 | 683 | 2.9 | 539 | 7.6 | 283 | 2.8 | 1511 | 3.0 | 427 | 2.9 |
| **Maternal disposable income**^a,c,d^ | | | | | | | | | | | | | | | | | | |
| 1^st^ quartile | 550 | 12.5 | 2772 | 13.4 | 815 | 14.7 | 610 | 24.3 | 3065 | 24.7 | 756 | 21.5 | 2631 | 25.6 | 12843 | 25.6 | 3636 | 25.0 |
| 2^nd^ quartile | 1012 | 22.9 | 4853 | 23.5 | 1208 | 21.8 | 613 | 24.4 | 3096 | 24.9 | 791 | 22.5 | 2419 | 23.5 | 12189 | 24.3 | 3270 | 22.5 |
| 3^rd^ quartile | 1396 | 31.6 | 6158 | 29.8 | 1701 | 30.7 | 623 | 24.8 | 3077 | 24.8 | 765 | 21.8 | 2489 | 24.2 | 12211 | 24.3 | 3373 | 23.2 |
| 4^th^ quartile | 1395 | 31.6 | 6293 | 30.5 | 1717 | 31.0 | 644 | 25.7 | 3069 | 24.7 | 978 | 27.8 | 2552 | 24.8 | 12039 | 24.0 | 3887 | 26.8 |
| Missing^a^ | 60 | 1.4 | 590 | 2.9 | 94 | 1.7 | 20 | 0.8 | 113 | 0.9 | 228 | 6.5 | 186 | 1.8 | 983 | 2.0 | 356 | 2.5 |
| **Paternal disposable income**^a,c,d^ | | | | | | | | | | | | | | | | | | |
| 1^st^ quartile | 731 | 16.6 | 3723 | 18.0 | 986 | 17.8 | 579 | 23.1 | 2968 | 23.9 | 694 | 19.7 | 2422 | 23.6 | 12201 | 24.3 | 3673 | 25.3 |
| 2^nd^ quartile | 1099 | 24.9 | 5137 | 24.9 | 1404 | 25.4 | 582 | 23.2 | 3029 | 24.4 | 775 | 22.0 | 2399 | 23.3 | 12319 | 24.5 | 3472 | 23.9 |
| 3^rd^ quartile | 1280 | 29.0 | 5517 | 26.7 | 1541 | 27.8 | 655 | 26.1 | 2977 | 24.0 | 915 | 26.0 | 2484 | 24.2 | 12282 | 24.4 | 3368 | 23.2 |
| 4^th^ quartile | 1192 | 27.0 | 5427 | 26.3 | 1477 | 26.7 | 616 | 24.5 | 2977 | 24.0 | 855 | 24.3 | 2650 | 25.8 | 11745 | 23.4 | 3529 | 24.3 |
| Missing^a^ | 111 | 2.5 | 862 | 4.2 | 127 | 2.3 | 78 | 3.1 | 469 | 3.8 | 305 | 8.7 | 322 | 3.1 | 1718 | 3.4 | 480 | 3.3 |
| **Average days/year with hospital contacts**^f^ | | | | | | | | | | | | | | | | | | |
|  | Average days/year | | Average days/year | | Average days/year | | Average days/year | | Average days/year | | Average days/year | | Average days/year | | Average days/year | | Average days/year | |
| 0-4 years after reference date | 15.1 |  | 0.7 |  | 0.8 |  | 20.6 |  | 0.9 |  | 1.1 |  | 18.5 |  | 0.7 |  | 0.8 |  |
| 5-9 years after reference date | 4.9 |  | 0.9 |  | 1.0 |  | 7.9 |  | 1.3 |  | 1.3 |  | 6.1 |  | 0.9 |  | 1.0 |  |
| 10-14 years after reference date | 3.3 |  | 1.1 |  | 1.4 |  | 4.7 |  | 1.6 |  | 1.7 |  | 3.7 |  | 1.1 |  | 1.3 |  |
| 15-19 years after reference date | 3.1 |  | 1.3 |  | 1.5 |  | 4.1 |  | 1.9 |  | 2.0 |  | 3.3 |  | 1.3 |  | 1.4 |  |
| 20-24 years after reference date | 2.9 |  | 1.4 |  | 1.7 |  | 4.9 |  | 1.9 |  | 2.3 |  | 2.9 |  | 1.4 |  | 1.6 |  |
| 25-29 years after reference date | 3.3 |  | 1.7 |  | 1.8 |  | 5.4 |  | 2.2 |  | 2.6 |  | 3.5 |  | 1.6 |  | 1.6 |  |
| 30-34 years after reference date | 4.4 |  | 1.6 |  | 1.8 |  | 6.1 |  | 2.0 |  | 2.5 |  | 3.8 |  | 1.7 |  | 1.6 |  |
| 35-39 years after reference date | 5.5 |  | 1.8 |  | 2.1 |  | 7.5 |  | 2.0 |  | 3.2 |  | 4.8 |  | 1.8 |  | 2.2 |  |
| **End event** | | | | | | | | | | | | | | | | | | |
| Death | 569 | 10.7 | 396 | 1.6 | 91 | 1.4 | 542 | 9.6 | 410 | 1.5 | 124 | 1.5 | 831 | 8.1 | 610 | 1.2 | 174 | 1.2 |
| Emigration (1^st^ emigration) | 391 | 7.3 | 2343 | 9.4 | 614 | 9.1 | 59 | 1.0 | 504 | 1.8 | 111 | 1.3 | 433 | 4.2 | 2759 | 5.5 | 801 | 5.5 |
| Last time identified in population register / lost to follow-up | 5 | 0.1 | 26 | 0.1 | 3 | <0.1 | 0 | 0 | 0 | 0 | 0 | 0 | 0 | 0 | 0 | 0 | 0 | 0 |
| End of study  DEN: 11 Aug 2017  SWE: 31 Dec 2016  FIN: 31 Dec 2014 | 4378 | 81.9 | 22268 | 89.0 | 6015 | 89.5 | 5071 | 89.4 | 27091 | 96.7 | 8164 | 97.2 | 9013 | 87.7 | 46896 | 93.3 | 13547 | 93.3 |
| **Median time of follow-up in years (range)** | 20.7  (5.0-46.6) |  | 21.8  (5.0-46.6) |  | 23.2  (5.0-46.6) |  | 19.1  (5.0-44.0) |  | 20.4  (5.0-44.0) |  | 21.1  (5.0-44.0) |  | 20.6  (5.0-46.0) |  | 21.8  (5.0-46.0) |  | 22.3  (5.0-46.0) |  |
| **Type of sibling^g^** | | | | | | | | | | | | | | | | | | |
| Full sibling | - | - | - | - | 5505 | 81.9 | - | - | - | - | 7351 | 87.5 | - | - | - | - | 11854 | 81.6 |
| Half sibling | - | - | - | - | 1218 | 18.1 | - | - | - | - | 1048 | 12.5 | - | - | - | - | 2668 | 18.4 |
| **Age difference between survivor and sibling** | | | | | | | | | | | | | | | | | | |
| Less than 5 years older | - | - | - | - | 2377 | 35.4 | - | - | - | - | 2754 | 32.8 | - | - | - | - | 4696 | 32.3 |
| 5-10 years older | - | - | - | - | 1299 | 19.3 | - | - | - | - | 1742 | 20.7 | - | - | - | - | 2968 | 20.4 |
| Less than 5 years younger | - | - | - | - | 1972 | 29.3 | - | - | - | - | 2539 | 30.2 | - | - | - | - | 4296 | 29.6 |
| 5-10 years younger | - | - | - | - | 1075 | 16.0 | - | - | - | - | 1364 | 16.2 | - | - | - | - | 2562 | 17.6 |

Reference date corresponds to the date of diagnosis for the population comparisons. For siblings the reference date corresponds to the date when a sibling was of the same age as the respective survivor at diagnosis.

^a^Information tied to the years of register coverage in the respective country (see Table 1). Parental socioeconomic information from Finland were tied to the years from 1980 onwards.

^b^Corresponds to reference year.

^c^Characteristics correspond to the biological parents.

^d^Corresponds to the year before reference year; if not available, then to the year closest to the year before reference year.

^e^Parents with missing education in Finland have been allocated to lowest education category, as only education from secondary level and above is registered in Finland.

^f^Average number of days/year with inpatient and outpatient hospital contact for any somatic and mental disorder during 5-year periods after reference date.

^g^Adoptive siblings from Sweden were assigned to the group of full siblings, whereas adoptive siblings from Denmark and Finland cannot be specifically identified and therefore may be found in both groups.
